# The Impact of Obesity on Mortality and Complications in Posterior Retroperitoneoscopic Adrenalectomy

**DOI:** 10.7759/cureus.42421

**Published:** 2023-07-25

**Authors:** Yi Th'ng Seow, Munyaradzi G Nyandoro, Shearn Poh, Yeow Chun Tee, Ming Khoon Yew, Sze Ling Wong

**Affiliations:** 1 General and Endocrine Surgery, Royal Perth Hospital, Perth, AUS; 2 General Surgery, Fiona Stanley Hospital, Perth, AUS; 3 General and Endocrine Surgery, St. John of God Murdoch Hospital, Murdoch, AUS

**Keywords:** endocrine surgery. gi surgery, conversion, complication, obese, adrenalectomy, retroperitoneoscopic

## Abstract

Background

Obesity is a global epidemic. It influences surgical technique, ergonomics, safety, and outcomes. However, there is a paucity of evidence of obesity-related impact in posterior retroperitoneoscopic adrenalectomy (PRA). This study compared perioperative outcomes of obese and non-obese participants undergoing PRA.

Methodology

This is a multi-center retrospective cohort study of elective PRA from March 2014 to December 2022. Patient demographics, surgical techniques, clinicopathological parameters, and outcomes, including overall complication rate, were analyzed using SPSS version 27 (IBM Corp., Armonk, NY, USA).

Results

Seventy-five patients underwent a PRA, of which 97.3% were completed retroperitoneoscopically. The overall complication rate was (9.3%), and on subgroup analysis, the obese cohort had a lower percentage complication profile at 6.5%. Male participants comprised 52%, with a median age of 55 (IQR=19). The median BMI was 29.0 (IQR=8), of which 41% were obese, and 40% were overweight.

Univariate analysis showed that being obese was not significantly associated with a higher complication rate (p=0.471). In addition, there was no significant increase in conversion (p=0.508), bleeding/transfusion (p=0.508), surgical site infection (SSI; p=1.000), incisional hernia (p=1.000), ICU or high dependency unit admission (p=0.292) and any-cause mortality (p=1.000). No sentinel deaths directly related to PRA were recorded. Procedure duration was longer in obese (117 mins) vs. non-obese participants (88.9 mins, p=0.022). However, there was no significant difference in the length-of-hospital stay (p=0.592). The cohort conversion rate was (2.7%), and tumor size was associated with a higher conversion rate (35.4 vs. 62.5mm, p=0.040).

Conclusion

Posterior retroperitoneoscopic adrenalectomy can be a safe procedure in obese populations, and obesity does not increase perioperative morbidity or mortality.

## Introduction

Laparoscopic adrenalectomy has been the gold standard technique for resecting benign adrenal tumors since its introduction in 1992 by Ganger in Canada [1] and Higashihara in Japan [2]. Early works by Brunt LM et al. [3] of insufflating the retroperitoneum with carbon dioxide in swine models facilitated retroperitoneal adrenalectomy in humans [4,5]. Laparoscopic surgery is associated with reduced blood loss, lower post-operative pain, and shorter hospital admission. Walz MK et al. further inspired the wide adoption of the posterior approach for small and benign adrenal lesions, as it provides a direct approach to the adrenal glands [6]. Obesity is a global epidemic on the rise, with a third of the world’s population classified as either overweight or obese [7]. It is widely accepted that obese patients are technically more challenging and carry an increased risk of intra- and post-operative surgical complications [8,9]. Intra-operative prone positioning of obese patients is a health and safety risk for the patient and theatre staff. Excess visceral fat limits the operative field, thus often making it difficult to identify critical structures and perform dissection. In addition, obesity is often associated with co-morbidities like diabetes, obstructive sleep apnoea, and hypertension, which make perioperative optimization challenging. There is a paucity of evidence describing how obesity impacts posterior retroperitoneoscopic adrenalectomy (PRA). This paper describes a multi-center experience in Western Australia, comparing perioperative outcomes of obese and non-obese participants undergoing a PRA. The primary aim was to determine the overall complication rate for PRA and evaluate contributing factors and associated morbidity.

## Materials and methods

Study population

All adult patients who underwent elective PRA surgery from March 2014 to December 2022 at two tertiary teaching institutions in Western Australia were retrospectively analyzed. Three fellowship-trained endocrine surgeons performed the procedures, and a cohort of 75 participants was identified. Relevant clinical and outcome data were collected from medical records, radiology, and pathology reports. Two investigators (Yi Th'ng Seow and Munyaradzi G. Nyandoro) independently recorded this data to ensure accuracy. All data were entered into a database using Microsoft® Excel for Mac 2020. BMI was categorized according to the WHO classification system as normal, overweight, and obese (18-24.9, 25.0-29.9, and >30.0 Kg/m2), respectively. These three were then re-categorized for dichotomous analysis as obese vs. non-obese groups. The Clavien-Dindo classification was used to categorize the complications, on which the overall complication rate was calculated. Surgical site infection (SSI) was defined as superficial or deep space infection with associated clinical, biochemical, microbiological, and imaging correlation. The incisional hernia was defined as clinical or radiological (computed tomography or ultrasound). Per therapeutic guidelines, unless there was a documented contra-indication, appropriate prophylactic antibiotic therapy was defined as a weight-adjusted dose of cephazolin given at induction or within 60 minutes of surgical incision. Operative time was defined as the time in minutes between the initial incision and closure of the surgical wound.

Statistical analysis**

Baseline characteristics and outcome data for each procedure group were described using mean (± SD), median, and IQR or frequencies/proportions (%), depending on the distribution. The nonparametric Mann-Whitney U test analyzed outcomes for continuous unpaired variables. Dichotomous results were compared between groups using χ2 or Fisher's exact tests with no adjustment for multiple comparisons. The univariate and multivariate analyses evaluated the relationship between the overall complication risk and other factors. Multivariate binary logistic regression was used to model conversion odds while adjusting for potential confounders, such as obesity, age, side of the lesion, and indication for surgery. The independent variables selected in the regression model were consistent with the current literature on risk factors. All analyses were performed using SPSS version 27 (IBM Corp., Armonk, NY, USA), and a two-tailed p-value of <0.05 was considered statistically significant.

Primary and secondary outcomes

The study's primary objective was to compare the overall complication rate between obese and non-obese participants. The secondary outcomes evaluated the relationship between obesity and operative time, total length of hospital admission, length of stay in the ICU, 30-day mortality rate, and 30-day hospital readmission rate. 

Procedure details 

Patient Positioning

**Figure 1:**
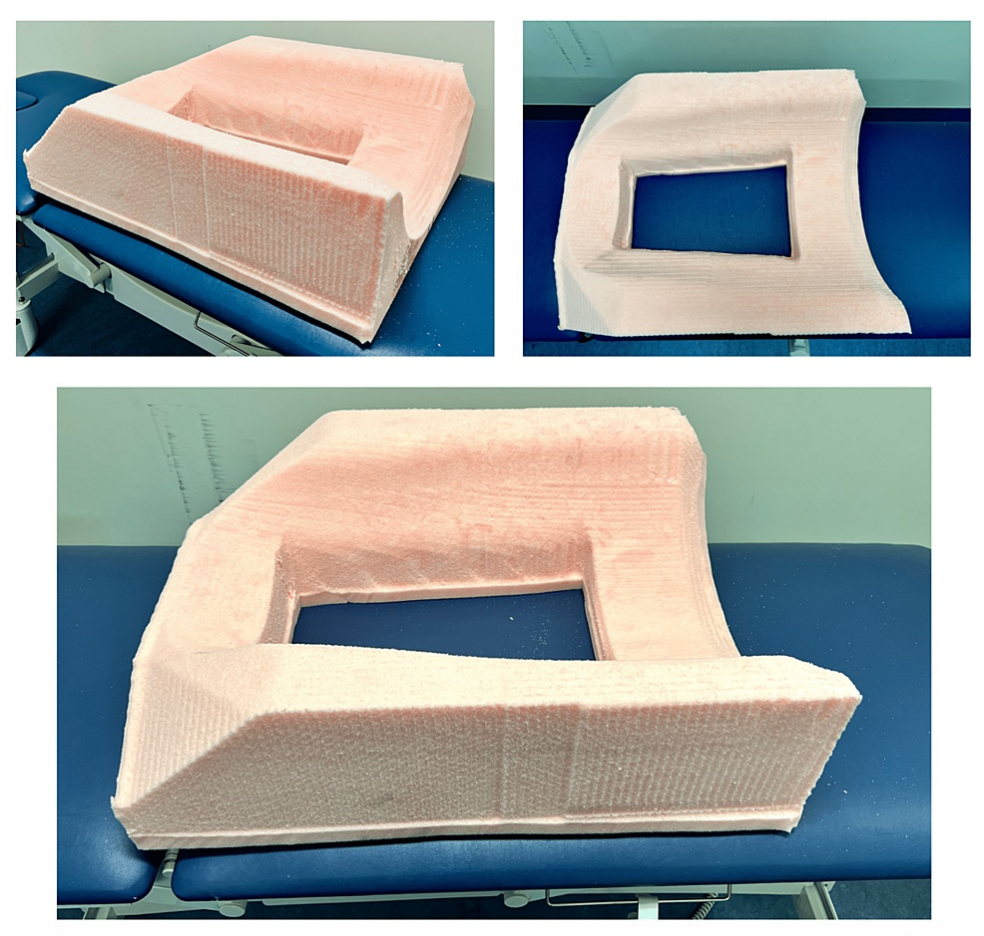
Novel customized polyurethane foam cushion for the patient.

The perioperative preparation was standardized per the WHO Surgical Safety checklist. After general anaesthesia, the patient was placed in a prone position, with hips flexed at a 90-degree angle, allowing maximization of the space between the ribs and iliac crests [8]. For non-obese patients, a gel cushion was used to support the hip and a pillow to support the chest, while for obese patients above 120 kg, a novel customized polyurethane foam cushion was utilized. Using a three-dimensional image of the patient's torso, the bioengineering department created a custom foam cushion to facilitate the prone positioning of the patients on the operating table (Figure 1). This innovation maximizes access, allows abdominal excursion during ventilation, and prevents pressure injury.

Port Placement

The first incision is made inferior to the tip of the 12th rib, and dissection is deepened with a combination of blunt and sharp dissection. A small retroperitoneal working space is created using blunt finger dissection, followed by the insertion of a 10 mm medial working port at the junction of the lateral border of the erector spinae muscle below the 12th rib; and a 5 mm lateral working port under the 11th rib. Both working port trocars are inserted under finger guidance and angled cranially at 45 degrees. The retroperitoneoscope is inserted via the middle port, and dissection through the retroperitoneal space through the Gerota's fascia is created using LigaSure™ (Medtronic, Dublin, Ireland), with insufflation pressures at 25-30 mmHg. The port placement is mirrored for bilateral cases. High insufflation pressure keeps the retroperitoneal space open and minimizes bleeding from small vessels [8,9].

Ethics

This project was approved as low risk by the Royal Perth and Fiona Stanley Hospitals Safety and Quality Improvement Governance Ethics sub-committees (Refs. #34956 and #49977).

## Results

All patient characteristics 

Over the study period, 75 participants underwent PRA. The clinicopathological characteristics of patients are shown in Table 1. Male participants comprised 52%, with a median age of 55 (IQR=19). The median BMI was 29.0 (IQR=8). The mean weight was 88.3 kg (SD±23.3). Most patients were ASA class II or III, accounting for 96.0% of the study participants. Forty-one percent of participants were obese, and forty percent of patients were overweight. Median procedure durations were 90.0 minutes, with a median length of stay in the hospital being two days (IQR=1). The HDU/ICU admission rate was 12%. The median imaging tumor size was 34.0 mm (IQR=24) (Table 1).

**Table 1:** Summary of participant characteristics. Values are the number (nn) of participants (%) unless otherwise indicated. * Mdn: Median, M: Mean; nn: number. ^†^ Body mass index, ^‡^ COPD: Chronic obstructive pulmonary disease, ^§^ ASA: American Association of Anaesthesiology; ^|| ^HDU/ICU: High Dependency Unit and Intensive Care Unit.

Variable		Number (n), Proportion (%)
Demographic characteristics	Male: Female n (%)	39 (52.0): 36 (48.0)
	Age (Years: Median, IQR) *	55 (19)
	BMI = Kg/m^2^ (Number: Median, IQR) ^†^	29.0 (8)
	Weight (Number: Mean ± SD)	88.3 ± 23.3
	Length of Hospital (Days: Median, IQR)	2.0 (1.0)
	Follow-up period (Months: Mean ± SD)	50.4 ± 26.8
		n (%)
Co-morbidities	Hypertension	31 (41.3)
	Obese	31 (41.3)
	Overweight	30 (40.0)
	Previous abdominal surgery	22 (29.3)
	Diabetes	18 (24.0)
	Smoker	16 (21.3)
	Normal weight	14 (18.7)
	Malignancy	13 (17.3)
	Respiratory disease (COPD) ^‡^	6 (8.0)
	Chronic kidney disease	4 (5.3)
Operation characteristics	Procedure duration (Minutes: Median, IQR) *	90.0 (49)
	Drain	4 (5.3)
Indication for surgery	Incidentaloma	34 (45.3)
	Conns	18 (24.0)
	Pheochromocytoma	10 (13.3)
	Metastasis	9 (12.0)
	Cushing’s	4 (5.3)
Specimen characteristics	Imaging size (millimetres: Median, IQR) *	34.0 (24)
	Histological size (millimetres: Median, IQR) *	50.0 (37)
Tumour side	Right	37 (49.3)
	Left	34 (45.3)
	Bilateral	4 (5.3)
ASA ^§^	One	1 (1.3)
	Two	37 (49.3)
	Three	35 (46.7)
	Four	2 (2.7)
Patient position	Jack-knife prone	75 (100)
HDU/ICU Admission ^||^	Yes	9 (12.0)
Antiplatelet agent	Yes	13 (17.3)
Anticoagulants	Yes	2 (2.7)
Sentinel event	Yes	0 (0.0)

Comparison of categorical participant characteristics by BMI group 

The demographics of the obese and non-obese subgroups were largely similar. The only statistically significant difference was in gender distribution, with a higher proportion of obese participants being female (55.6% vs. 28.2%). There was also a higher concurrent association between obesity and other comorbidities, such as hypertension and chronic respiratory conditions; however, this did not reach statistical significance (Table 2).

**Table 2:** Characteristics of retroperitoneoscopic adrenalectomy patients and univariate chi-square* results for independent variables, with obesity as the dependent variable. Values are the number of participants (%) unless otherwise indicated. *Pearson Chi-Square analysis, and Fisher’s Exact Test (for cell values <5), and * Bolded denotes significance at p<0.05 ^†^ ASA: American Association of Anaesthesiology (score), ^‡^ COPD: Chronic obstructive pulmonary disease.

Variable (N = 75)		Obese Subgroup Population (n=31)		
		(n)	(%)	P-value
Age (years)	60 years and younger	18	41.9	0.914
	61 years and older	13	40.6	
Gender	Male	11	28.2	0.016 *
	Female	20	55.6	
ASA score ^†^	One	0	0.0	0.288
	Two	14	37.8	
	Three	15	42.9	
	Four	2	100	
Drain	No	31	43.7	0.084
	Yes	0	0.0	
Laterality of lesion	Unilateral	28	39.4	0.300
	Bilateral	3	75.0	
Antiplatelets	No	25	40.3	0.698
	Yes	6	46.2	
Smoker	No	24	40.7	0.825
	Yes	7	43.8	
Hypertension	No	9	29.0	0.096
	Yes	22	50.0	
Diabetic	No	23	40.4	0.758
	Yes	8	44.4	
Respiratory disease (COPD) ^‡^	No	27	39.1	0.224
	Yes	4	66.7	
Chronic Kidney Disease	No	30	42.3	0.638
	Yes	1	25.0	
Malignancy	No	28	45.2	0.216
	Yes	3	23.1	
Previous abdominal surgery	No	20	37.7	0.326
	Yes	11	50.0	

Comparison of continuous participant characteristics by BMI group

Variables such as age, ASA class, and tumour size (both on imaging and histopathology) were evenly distributed between the obese and non-obese subgroups. There was a statistically significant difference in operation duration between obese and non-obese participants (117.7 vs. 88.9 minutes, p=0.022). However, there was no significant difference in the length of hospital stay (2.6 vs. 2.8 days, p=0.592) (Table 3).

**Table 3:** Comparison of continuous characteristics for retroperitoneoscopic participants with obesity as the dependent variable Values are the number of participants (%) unless otherwise indicated. Nonparametric independent-samples Mann-Whitney U test and * Bolded denotes significance at p<0.05 ^† ^Body Mass Index, ^‡ ^ASA: American Association of Anaesthesiology (score), ^§^ LoHS: Length of hospital stay.

Variable	Obese	Non-obese	P-value
Age (years)	55.2	56.7	0.590
BMI = kg/m^2^ (nn) ^†^	36.6	26.3	<0.001*
Weight (kg)	104.9	76.6	<0.001*
ASA (nn) ^‡^	2.6	2.4	0.267
Initial LoHS (days) ^§^	2.6	2.8	0.592
Procedure duration (mins)	117.7	88.9	0.022*
Tumour imaging size (mm)	35.1	36.8	0.675
Tumour histological size (mm)	51.3	50.8	0.936
Follow-up period (months)	36.1	60.2	<0.001*
Follow-up period (years)	2.7	4.6	<0.001*

Summary of all post-operative complications**

The overall complication rate for the cohort was 9.3%. Class I complications, according to the Clavien-Dindo classification, were the most frequent, at 4.0%. There was a low transfusion rate of 2.7%. There were no procedure-specific related deaths. Both the representation and readmission rates were low, at 2.7% and 1.3% respectively (Table 4).

**Table 4:** Summary of postoperative complications – combined (inpatient and outpatient). Values are the number of participants (%) unless otherwise indicated. ^† ^HDU/ICU: High Dependency Unit and Intensive Care Unit, ^‡ ^Sentinel death: Directly related to surgical procedure, ^§ ^Delayed: Being representation beyond the initial 30-day follow-up.

Variable		Number (Proportion)
N = 75		n (%)
Postoperative outcomes	ICU/HDU admission ^†^	9 (12.0)
	Anytime complication	7 (9.3)
	Any cause mortality	5 (6.7)
	Reposition Prone to Supine	3 (4.0)
	Conversion to open	2 (2.7)
	Bleeding /Transfusion	2 (2.7)
	Incisional hernia	2 (2.7)
	Surgical site infection	1 (1.3)
	Sentinel death ^‡^	0 (0.0)
	30-day representation rate	2 (2.7)
	30-day readmission rate	1 (1.3)
	Delayed representation ^§^	1 (1.3)
	Clavien-Dindo Classification	
	I	3 (4.0)
	II	2 (2.7)
	III	2 (2.7)

Obese participant's morbidity profile and outcomes​​​​​​​

No statistically significant associations were found between obesity and post-operative complications. The overall risk of complications at any time for the obese group was 6.5% (p=0.471). None of the operations on obese participants were converted to open procedures (p=0.508). Additionally, there was no increased risk for unplanned HDU/ICU admission among this group (p=0.292) (Table 5).

**Table 5:** Obese participant's morbidity profile and univariate analysis outcomes post retroperitoneoscopic adrenalectomy. Values are the number of participants (%) unless otherwise indicated. *Pearson Chi-Square analysis, and Fisher’s Exact Test (for cell values <5), and * Bolded denotes significance at p<0.05 ^†^ HDU/ICU: High Dependency Unit and Intensive Care Unit, ^‡^ Cause of death: Not directly related to surgical procedure and not occurring within the first three months.

Variable (N = 75)		Obese subgroup population (n=31)		
		(n)	(%)	P-value
Any Complication	No	5	11.4	0.471
	Yes	2	6.5	
Reposition from prone to supine	No	3	6.8	0.263
	Yes	0	0.0	
Conversion to open	No	2	4.5	0.508
	Yes	0	0.0	
Bleeding/Transfusion	No	2	4.5	0.508
	Yes	0	0.0	
Surgical site infection	No	1	2.3	1.000
	Yes	0	0.0	
Incisional hernia	No	1	2.3	1.000
	Yes	1	3.2	
ICU/HDU admission ^†^	No	7	15.9	0.292
	Yes	2	6.5	
Any cause mortality ^‡^	No	3	6.8	1.000
	Yes	2	6.5	

Risk profile for any complication: univariate and nonparametric analysis

The overall complication rate was 9.3% (n=7). Non-parametric analysis revealed significant associations between the overall complication risk and imaging tumour size, with tumours larger than 60mm associated with a higher complication rate (33.4 vs. 61.9mm, p=0.003). Interestingly, a higher incidence of complications was observed in younger participants (43.9 vs. 57.4 years, p=0.016). Complications were also significantly associated with longer hospital stays (2.5 vs. 4.3 days, p=0.001) and longer procedure durations (95.5 vs. 152.7 minutes, p=0.044) (Table 6).

**Table 6:** Comparison of characteristics for retroperitoneoscopic participants based on continuous variables, with overall complication rate as the dependent variable. Values represent the number (nn) of participants (%), unless otherwise specified. Statistical analyses include the nonparametric independent-samples Mann Whitney U test, Pearson Chi-Square analysis, and Fisher’s Exact Test (for cell values <5). Results indicated by an asterisk (*) are bolded to denote statistical significance at p<0.05. ^†^ Body Mass Index; ^‡^ ASA: American Association of Anaesthesiology (score);^ §^ LoHS: Length of hospital stay.

Variable (N = 75)	Any Complication		P-value
	Yes	No	
Age (years)	43.9	57.4	0.016*
BMI Kg/m^2^ (nn) ^†^	29.0	30.7	0.662
Weight (kg)	87.3	88.4	0.935
ASA (nn) ^‡^	2.4	2.5	0.710
Initial LoHS (days) ^§^	4.3	2.5	0.001*
Procedure duration (mins)	152.7	95.5	0.044*
Tumour imaging size (mm)	61.9	33.4	0.003*
Tumour histological size (mm)	75.9	48.5	0.017*

Neither the side of the lesion (p=0.699) nor the use of anticoagulants or antiplatelets (p=1.000) was associated with a higher complication rate. Similarly, being a smoker (p=0.637), having hypertension (p=0.118), diabetes (p=1.000), COPD (p=0.456), or CKD (p=1.000) did not correlate with higher complication rates. The indication for surgery (p=0.180) and the final histopathological diagnosis (p=0.092) were not statistically significant factors related to complications (Table 7).

**Table 7:** Comparison of characteristics of categorical variables among retroperitoneoscopic participants, with the overall complication rate being the dependent variable. Values are represented as the number of participants (%) unless otherwise indicated. Analyses were conducted using nonparametric independent-samples Mann-Whitney U test, Pearson Chi-Square analysis, and Fisher’s Exact Test (for cell values <5). Entries in bold* denote significance at p<0.05. ^†^ COPD: Chronic obstructive pulmonary disease.

		Any Complication		P-value
		(n)	(%)	
Age (years)	60 years and younger	6	14.0	0.227
	61 years and older	1	3.1	
Gender	Male	6	15.4	0.109
	Female	1	2.8	
Laterality	Unilateral	7	9.9	1.000
	Bilateral	0	0.0	
Lesion side	Right	3	8.1	0.699
	Left	4	11.8	
	Bilateral	0	0.0	
Respiratory disease (COPD) ^||^	No	6	8.7	0.456
	Yes	1	16.7	
Chronic Kidney Disease	No	7	9.9	1.000
	Yes	0	0.0	
Smoker	No	5	8.5	0.637
	Yes	2	12.5	
Hypertension	No	5	16.1	0.118
	Yes	2	4.5	
Diabetic	No	6	10.5	1.000
	Yes	1	5.6	
Malignancy	No	6	9.7	1.000
	Yes	1	7.7	
Obese	No	5	11.4	0.693
	Yes	2	6.5	

Risk factors for conversion to open**

The overall conversion rate was 2.7% (n=2). Univariate analysis showed significant positive associations between the risk of conversion to open and the imaging tumor size, with tumors> 60 mm associated with a higher conversion rate (35.4 vs. 62.5mm, p=0.040) (Table 7). Conversion to open was also significantly associated with the indication for surgery; pheochromocytomas were more likely to result in a conversion (20% vs. 0%) (p=0.010). Conversion to open surgery was associated with a higher rate of HDU/ICU admission (1.5% vs. 11.1%), but this was not statistically significant (p=0.227). Similarly, higher transfusion rates were observed in conversions (1.4% vs. 50%; p=0.050). However, obesity and overweight were not associated with higher conversion rates (p=0.333, p=0.525) (Table 8).

**Table 8:** Comparison of continuous characteristics for retroperitoneoscopic participants with conversion to open as the dependent variable. Unless otherwise specified, values are given as the number of participants (%). Statistical significance at p<0.05 is denoted by bolded text. Nonparametric independent-sample comparisons were conducted using the Mann-Whitney U test. ^†^ Body Mass Index, ^‡^ ASA: American Association of Anaesthesiology (score), ^§^ LoHS: Length of hospital stay.

Variable (N = 75)	Conversion to open		P-value
	Yes	No	
Age (years)	35.0	56.7	0.014*
BMI Kg/m^2^ (nn) ^†^	24.7	30.7	0.333
Weight (kg)	77.5	88.6	0.525
ASA (nn) ^‡^	2.0	2.5	0.274
Initial LoHS (days) ^§^	4.5	2.6	0.087
Procedure duration (mins)	200.0	98.1	0.035*
Tumor imaging size (mm)	62.5	35.4	0.040*
Tumor histological size (mm)	70.0	50.5	0.162

## Discussion

Operative techniques 

In recent years, minimally invasive adrenalectomy has become the gold standard for the surgical treatment of most adrenal lesions. The two most common surgical approaches are laparoscopic transperitoneal adrenalectomy (LTA) and PRA. The LTA approach provides a familiar anatomy entry, but the mobilization of intraperitoneal structures, e.g., colon, liver, and spleen, is often necessary to access the adrenal glands. It can also be more technically challenging in patients with previous abdominal surgeries because of abdominal adhesions [10]. PRA has gained popularity because it avoids entry into the peritoneal cavity and provides direct access to the adrenal glands, though it does present a steep learning curve for surgeons. Kook Y et al. have summarized that PRA is a favorable surgical method, demonstrating similar surgical outcomes to conventional LTA [11], but with significantly shorter operation times, quicker diet initiation times, fewer hospital readmissions, and less need for analgesia than LTA. Therefore, in the absence of any contraindications, this method is the preferred and most widely used technique at our study center. 

Obesity endemic factors 

According to Australia's latest National Health Survey, two-thirds of adults were overweight or obese. Obesity creates numerous anesthetic and surgical challenges in abdominal surgery [9]. However, the effect of obesity on the outcome of patients after PRA surgery is unknown. Studies have reported that high BMI is an independent risk factor for peri-renal fat adhesions, which could predispose to more difficult renal dissection and result in bleeding and decapsulation [12, 13]. The initial concern of higher complication risk was raised by Walz MK et al., who pioneered PRA and did not recommend the retroperitoneal approach for patients with BMI >45kg/m2 [14]. Walz MK postulated that the intraperitoneal viscera could compress the retroperitoneum space in the prone position in morbidly obese patients. 

Previous surgical literature has discouraged the performance of PRA in obese patients [6]. However, recent studies have described the feasibility and safety of PRA in the obese population [15-22]. Hu Q et al. reported PRA as a safe operation in obese patients but used a lateral decubitus position instead of a jack-knife position [16]. In this study, a multidisciplinary team approach was adopted for preoperative optimization. The expert biomedical engineering team custom-made the abdominal and chest cushion foams using three-dimensional technology to allow abdominal excursion during ventilation and decrease pulmonary splinting [18].

In this series, obesity was associated with increased procedure duration, consistent with other reported literature. However, the increased operative time did not correlate with higher morbidity and complication rates in the obese group. The increased surgical time (skin-to-skin) was independent of the time related to surgical positioning. It has been postulated that the technical challenges of adrenalectomy in obese patients, closely associated with the complex dissection planes due to increased retroperitoneal fat mass, might help explain the longer operation durations reported in these patient groups. Recent studies have suggested that an assessment of visceral fat using CT scans can predict surgical outcomes [23].

Complications 

The overall complication rate in this study was 9.3%, while it was even lower for the obese sub-group at 6.5%. The low morbidity observed is consistent with literature-reported profiles between 0 and 14.4% [14]. Retroperitoneal adrenalectomy-associated complications include pleural tears, pneumothorax, SSI, bleeding, hypoesthesia, and abdominal wall relaxation. This study reported single cases of abdominal wall hematoma and SSI, which were managed conservatively. An isolated incident of significant intra-operative bleeding required a blood transfusion. The incisional hernia occurrences were surgically repaired without further complications. No sentinel deaths directly related to PRA were recorded. Extra-adrenal metastatic disease was the lead cause of all-cause mortality post metastasectomy. No significant differences were observed between the obese and non-obese populations regarding perioperative complications. Most of the complications were either Clavien-Dindo classification I or II.

Contrary to literature reports on obesity being a risk factor for surgical site occurrences and perioperative morbidity, [24] this study did not find a significantly increased risk of perioperative morbidity and complications such as SSI, incisional hernia, bleeding risk, or unplanned admission to HDU/ICU, 30-day representations and readmissions. Identifying key predictive perioperative risk factors for the likelihood of any complication could improve preoperative planning and optimization. This individualized approach will foster a sustainable, efficient healthcare delivery model and good patient-related outcomes.

Conversion factors 

Recent studies have reported a conversion rate of PRA between 1.7 and 18% [14, 25-27]. This study's conversion rate was low, and it occurred in the non-obese group, confirming that obesity is not significantly associated with a higher conversion rate. The conversions were due to intricate dissection of the left adrenal lesions, an observation reported in the literature. They postulated that the left adrenal is technically more challenging due to its anatomical location. Still, this study did not find any statistical significance for conversion rate. These lesions were both pheochromocytomas on histopathology, with one only diagnosed post-operatively. The higher PRA pheochromocytomas-related conversion rates observed in this study contradicted findings reported by Hisano M et al. [28] and Lei K et al. [29]; this could be due to bias, given this study's low overall conversion rate. Some studies have suggested that the measurement of retroperitoneal fat mass in preoperative CT can predict conversion rate [30].

Strengths and limitations

Limitations of this paper include its retrospective design and associated data collection issues; notably, there needed to be more complete data regarding follow-up and why some patients did not attend follow-up clinics. This study could have underestimated the long-term morbidity rate if the patients chose to present elsewhere to manage their complications. This study reported low events, meaning it was underpowered to make a conclusive statistical assessment of the complication subgroups. It is widely accepted that technical-related complications are usually encountered early in implementing a new technique due to the initial steep learning curve. Therefore, it can be reasonably extrapolated that given the low and encouraging complication rates presented in the paper during the early phases, technical complications will likely remain low as fellow-trained surgeons continue improving their expertise and efficiency in PRA. Despite these limitations, a comprehensive multi-database review was conducted with multi-source triangulation of data points using explicit definitions of variables and complications to allow for reproducibility. The authors plan on conducting a follow-up study to report on long-term outcomes.

## Conclusions

Posterior retroperitoneoscopic adrenalectomy can be a safe procedure in obese populations, and obesity does not increase perioperative morbidity or mortality.
